# Heterologous expression of interferon-stimulated genes reveals conserved anti-*Toxoplasma* properties between human and porcine cells

**DOI:** 10.3389/fimmu.2026.1790284

**Published:** 2026-04-02

**Authors:** Marzuq A. Ungogo, Praveena R. G. Chandrasegaran, Hamza Khalid, Jessica Powell, Hamna Jamil, Finn Grey, Musa A. Hassan

**Affiliations:** The Roslin Institute, Royal (Dick) School of Veterinary Studies, University of Edinburgh, Edinburgh, United Kingdom

**Keywords:** host-pathogen interaction, IDO1, interferon, IRF1, *Toxoplasma gondii*

## Abstract

*Toxoplasma* is a zoonotic parasite that can cause life-threatening illness in humans and animals. The similarities in *Toxoplasma* pathogenesis between pigs and humans make pigs an attractive model for studying human toxoplasmosis. However, the mechanisms underpinning these similarities remain poorly understood. Since interferon-gamma (IFNγ) is crucial for immunity to *Toxoplasma*, we hypothesized that IFNγ signaling pathways conserved between humans and pigs contribute to comparable *Toxoplasma* pathogenesis. Using a genetic gain-of-function strategy, we observed that human or porcine interferon regulatory factor 1 (IRF1) and indoleamine 2,3-dioxygenase 1 (IDO1) inhibited *Toxoplasma* replication in both homologous and heterologous human and porcine cells. IRF1-depleted porcine cells that failed to control *Toxoplasma* growth could be rescued by ectopic expression of human IRF1, which induces genes similar to those regulated by porcine IRF1. The expression of either human or pig IRF1 dampened *Toxoplasma-*induced transcriptional remodeling of porcine genes. Additionally, blocking IDO1 using 1-methyl tryptophan reversed parasite growth inhibition in cells expressing heterologous or homologous IDO1. These findings highlight conserved IFN-induced anti-*Toxoplasma* mechanisms between pigs and humans and enhance the potential of pigs as large animal models for human toxoplasmosis.

## 1 Introduction

*Toxoplasma gondii* is a zoonotic apicomplexan parasite that infects all warm-blooded vertebrates, including over a billion people worldwide, and can cause severe illness and mortality in humans and animals. Although not yet proven, *Toxoplasma* is also linked to mental and behavioural disorders, such as schizophrenia and autism (1–3). Pigs are particularly susceptible to *Toxoplasma* infection. Acute toxoplasmosis can cause severe morbidity and up to 57% mortality in piglets, while chronic *Toxoplasma* infection suppresses immunity and lowers productivity (4). Additionally, pork from chronically infected pigs presents a major risk for *Toxoplasma* transmission to humans (5).

In vertebrates, the interferon-gamma (IFN-γ) is essential for effective immunity against *Toxoplasma*, acting primarily through the induction of hundreds of interferon-stimulated genes (ISGs) (6). Lower levels of IFNγ in immunocompromised patients increases the risk of reactivation of chronic toxoplasmosis (7), which can result in fatal outcomes. In mice, the inhibition of IFNγ-induced responses either through the deletion of the IFNγ receptors or the effectors such as Signal Transducer and Activator of Transcription 1 (STAT1) leads to increased susceptibility to acute *Toxoplasma* infection (8, 9). Studies on IFNγ-responses to *Toxoplasma*, mostly in rodents, have generated insights into cell-autonomous immunity against the parasite. However, there are several differences in IFNγ-mediated control of *Toxoplasma* between rodents and humans, including the vital role of IFN-induced immunity related GTPases (IRGs) in resistance to *Toxoplasma* in rodents (10, 11), but not humans. Therefore, there is a need for better animal models for human toxoplasmosis.

There are several similarities between *Toxoplasma* pathogenesis in humans and pigs. Unlike in mice, postnatal *Toxoplasma* exposure in immunocompetent humans and pigs usually results in subclinical or mild illness (12, 13). Additionally, in both pigs and humans, intrauterine transmission may lead to abortion and congenital infection depending on gestational age (14–17), whereas in mice, such outcomes depend on immune status and genetic susceptibility (18, 19). These similarities suggest that *Toxoplasma* pathogenesis, including major immune responses, is conserved between humans and pigs, and highlight the potential of pigs as a model for human toxoplasmosis. However, the mechanism underlying these similarities are largely equivocal.

IFNγ binding to its receptors and subsequent activation of downstream effectors, such as Janus Kinase (JAK) and STAT, appear to be highly conserved in mammals (20, 21). However, the downstream genes stimulated by an activated STAT dimer or other IFNγ-induced transcriptional regulators have shown diversity among species (21, 22). Therefore, similarities in IFNγ-mediated responses to *Toxoplasma* between species will likely depend on mechanisms triggered directly or indirectly by functionally conserved ISGs, which may also elicit similar corresponding parasite response and defence. In this study, we used expression of orthologous ISGs in porcine and human cells to investigate whether functionally conserved ISGs modulate similar *Toxoplasma* pathogenesis in porcine and human cells. We report that human and porcine orthologues of interferon regulatory factor 1 (IRF1) and indoleamine 2,3-dioxygenase 1 (IDO1) can restrict *Toxoplasma* in both *cis*- and *trans*-species cell lines through conserved mechanisms.

## 2 Materials and method

### 2.1 Cell and parasite culture

Human foreskin fibroblasts (HFF), human embryonic kidney (HEK), HeLa, CACO-2, Intestinal porcine epithelial cells J2 (IPEC-J2) and neonatal swine kidney (NSK) cells were grown in DMEM supplemented with 10% v/v foetal bovine serum (FBS), 1% Glutamax^®^ (Gibco) and 25 U/ml penicillin/streptomycin. Transduced CACO-2 and NSK cells were selected by culturing in medium supplemented with 10 µg/mL and 2 µg/mL puromycin, respectively. All cell lines were maintained at 5% CO_2_ and 37 °C.

*Toxoplasma* type I RH Δhxgprt cLUCGFP (RH1–1 clone) and type II Pru Δhxgprt fLUCGFP (PruA7) strain each expressing GFP and firefly luciferase (23), were maintained through serial passage on human foreskin fibroblasts (HFF) monolayer. For experimental infection, flask of infected-HFF was washed in PBS to remove naturally egressed parasites, followed by scraping of the cells. Parasites were mechanically egressed by passage through 25 g needle, and then purified from host cell debris using 5.0 μm filter (Millex). Filtrate containing parasites was centrifuged at 1,700 *g* for 7 min, and the pellet resuspended in fresh complete medium at required density.

### 2.2 Plasmid and cloning

psCRPSY lentiviral plasmids (24) expressing tag-RFP and individual cDNA for human GBP2, IDO1, IRF1 and CCL2 genes were kind gifts from Sam Wilson (University of Glasgow). The open reading frames of porcine GBP2 (ENSSSCT00000007583), IDO1 (ENSSSCT00000007675), IRF1 (ENSSSCT00000015600) and CCL2 (ENSSSCT00000019290) were synthesised and cloned into the *NotI* and *XhoI* sites of psCRYPSY plasmid. In order to generate plasmids to knockout individual genes, sgRNA targeting each gene was selected from pig GeCKO library (25). IDO1 sgRNA was synthesized as complimentary oligo pair carrying *BsmB*I restriction sites (forward; caccgACCCCCAAGAAGTTCGCTGG, and reverse; aaacCCAGCGAACTTCTTGGGGGTc), annealed and ligated into *BsmBI*-digested lentiCRISPRv2 vector (Addgene, # 52961) expressing cas9 (26, 27). IRF1 knockout plasmid was generated by ligating annealed oligo pairs (forward; caccgATTGAACAGGCCCTTACTCC, and reverse; aaacGGAGTAAGGGCCTGTTCAATc) into *BbsI*-digested pKLV2-U6gRNA3(BbsI)-PGKpuro2ABFP vector (28). All plasmids were transformed into competent *E. coli* and single colonies were selected and verified for correct integration of each insert using Sanger sequencing.

### 2.3 Lentivirus production and transduction

Plasmids were packaged into lentivirus as previously described (29). Briefly, 5×10^6^ cells HEK 293T cells were seeded in T75 flask and incubated for 24 h. Constructed plasmid was mixed with lentiviral pMD2.G and PAX2 plasmids and co-transfected into the cells using Lipofectamine 2000 (Invitrogen) in OptiMem reduced serum medium without phenol red (Gibco). The medium was removed and replaced with fresh complete DMEM at 24 h post-transfection, and viral supernatant was collected at 48 h post-transfection. The supernatant containing the virus was passed through 0.45 μm filter, concentrated using PEG-it viral concentration solution (Systems Biosciences), and titrated to achieve >50% MOI during transduction. The WT CACO-2 and NSK cells were seeded for 24 h and transduced by the addition of lentivirus expressing ISGs and 16 μg/mL dextran followed by 72 h incubation (30). NSK IDO1 KO cells were generated by transducing WT NSK with lentivirus expressing cas9/sgRNA plasmid, whereas lentivirus expressing sgRNA only plasmid was used to transduce cas9-expressing NSK cells to generate IRF1 KO. The transduced cells were harvested and selected in puromycin 2 μg/ml (NSK) or 10 μg/mL (CACO-2) for 10–14 days, and single clones were generated by limiting dilution.

### 2.4 *Toxoplasma* infection

For the determination of parasite growth in CACO-2 and NSK cells expressing human or pig ISGs, 2.5×10^4^ cells were seeded in a 96-well plate 24 hours prior to infection. In order to determine the effect of IFNγ stimulation on parasite growth in WT and IDO1 KO mutants, 1.2×10^4^ cells were seeded in a 96-well plate for 24 h, and treated with human or pig IFNγ for another 24 hours prior to infection. To infect cells, media was removed and replaced with parasite suspension adjusted to MOI of 0.2. The plate was centrifuged at 250 g for 3 min, left on the bench for 5 min, and then incubated at 37 °C for 1 h to synchronise infection (31). Supernatant was then removed from the plate and replaced with fresh complete medium, followed by further incubation for 48 h or 72 h.

In order to inhibit IDO1-mediated IFNγ control of *Toxoplasma*, WT cells were pre-treated with 1 mM 1-methyl L-tryptophan (1-MT) for 1 h before IFNγ treatment or supplemented with 10 mM L-tryptophan 1 h post-infection (32). The cells expressing IDO1 were pre-treated with 1-MT 24 h pre-infection or supplemented with 1 mM L-tryptophan at 1 h post-infection.

### 2.5 Luciferase assay for determination of parasite growth

Luciferase activity assay as a proxy for parasite burden in infected cells was carried out as described previously (33) with minor modifications. Briefly, media was removed from infected cells in a 96-well plate, and the cells were rinsed twice with PBS to remove cell debris. Cells were then treated with 20 µL 1× lysis buffer (Promega) supplemented with proteinase inhibitor and incubated for 15 min at room temperature under gentle shaking. The plate was incubated at 37 °C for 10 minutes in Cytation 3 luminometer for 37 °C, followed by injection of 100 μL/well of Luciferase Assay Reagent (Promega), a 2 *s* delay and luminescence reading. A cycle of injection, delay and measurement was repeated for each well. Luminescence reading was normalised and reported as fraction of reading in infected WT cells.

### 2.6 RNA-sequencing sample preparation and analysis

NSK IRF1-/- cells were seeded in 6-well plates overnight and transduced in triplicates with lentivirus expressing psCRPSY empty vector, psCRPSY.hIRF1 or psCRPSY.pIRF1 for 48 h. Transduced cells were then harvested, seeded into fresh 6-well plates and incubated for 24 h. Cells were either left uninfected or infected with *Toxoplasma* type II PruA7 at MOI of 0.5 for 48 h. RNA was isolated from each sample using Qiagen RNeasy Mini Kit according to the manufacturer’s protocol, and RNA quality was assessed on Agilent 2100 Electrophoresis Bioanalyser (Agilent Technologies Inc) to have a RIN>8.0. Libraries were prepared using the NEBNEXT Ultra II Directional RNA Library Prep kit (NEB) and the Poly-A mRNA magnetic isolation module (NEB) according to the provided protocol. Poly-A containing mRNA molecules were purified, fragmented and reverse transcribed into cDNA and adapters were ligated to the fragments. Purified, adapter-ligated DNA was then multiplexed and sequenced on NextSeq 2000 platform (Illumina Inc) using the NextSeq 2000 P3 XLEAP-SBS Reagent Kit (100 cycles) to yield ~60 million 50 bp high quality strand-specific paired reads per sample. Reads were pseudoaligned to the Ensembl porcine and *Toxoplasma* genomes using Kallisto v0.51.1 (34) with 100 bootstraps to generate transcript-level expression estimates as transcripts per million (TPM). Gene-level differential expression analysis was performed using EdgeR as previously described (35). Gene Ontology enrichment analysis was carried out for differentially expressed genes (FDR < 0.05 and logFC > 2) using ClusterProfiler (36).

## 3 Results

### 3.1 Interferon-γ-receptor interaction is not conserved between pigs and humans

**Figure 1:**
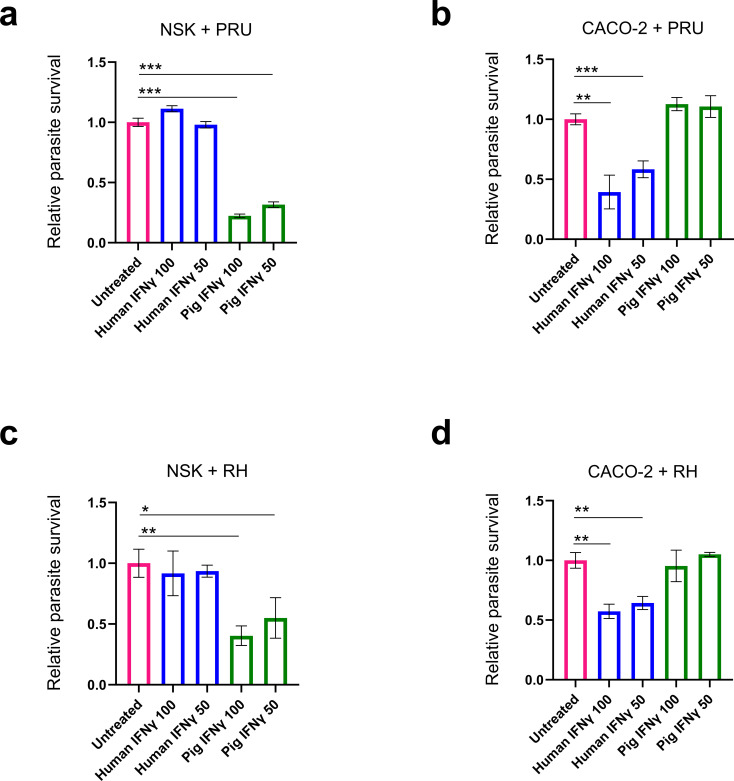
Cross-reactivity of porcine and human cells to exogenous IFNγ. **(a–d)** NSK **(a, c)** and CACO-2 **(b, d)** cells were stimulated with indicated concentrations of human (U/mL) or porcine IFNγ (ng/mL) for 24 h. Cells were then infected with either *Toxoplasma* PruA7 **(a, b)** or RH1-1 **(c, d)** at MOI of 0.2 for 72 h or 48 h, respectively. Infected cells were lysed and luciferase activity was measured in each group using Cytation 3 imaging station. Recorded luminescence reading in each group was normalised to untreated control as relative parasite survival. Data shows average (n=3, SEM as error bars) of one representative experiment out of 3 biological repeats showing similar trend. *p<0.05; p<0.005; *p<0.0005; and ns, not significant by unpaired t-test.

Given that pigs and humans have similarities in over 80% of immune parameters assayed (37), and IFNγ plays a critical role in *Toxoplasma* pathogenesis in both species (6), we reasoned that comparable *Toxoplasma* infection outcomes between the two hosts result from functional conservation of genes in the IFNγ signalling pathway. Thus, we first tested the cross-species activity of IFNγ by stimulating neonatal swine kidney (NSK) or human colorectal adenocarcinoma (CACO-2) cells with recombinant human or porcine IFNγ followed by infection with a type II *Toxoplasma* PRU strain expressing GFP and firefly luciferase (PruA7), which is highly sensitive to IFNγ. Although recombinant porcine IFNγ restricted *Toxoplasma* growth in porcine NSK (**Figure 1a**), it showed no effect on *Toxoplasma* growth in human CACO-2 cells (**Figure 1b**). Similarly, recombinant human IFNγ inhibited *Toxoplasma* in human CACO-2 (**Figure 1b**) but not in porcine NSK cells (**Figure 1a**). Similar patterns were observed against type I *Toxoplasma* strain RH1-1 (**Figures 1c, d**) and in other human and porcine cell lines (**Supplementary Figure 1**). Together, these observations indicate that similar porcine and human toxoplasmosis pathogenesis is not due to functional conservation at the level of IFNγ or its receptors.

### 3.2 A mini gain-of-function screen identifies ISGs that control *Toxoplasma* in both human and porcine cells

**Figure 2:**
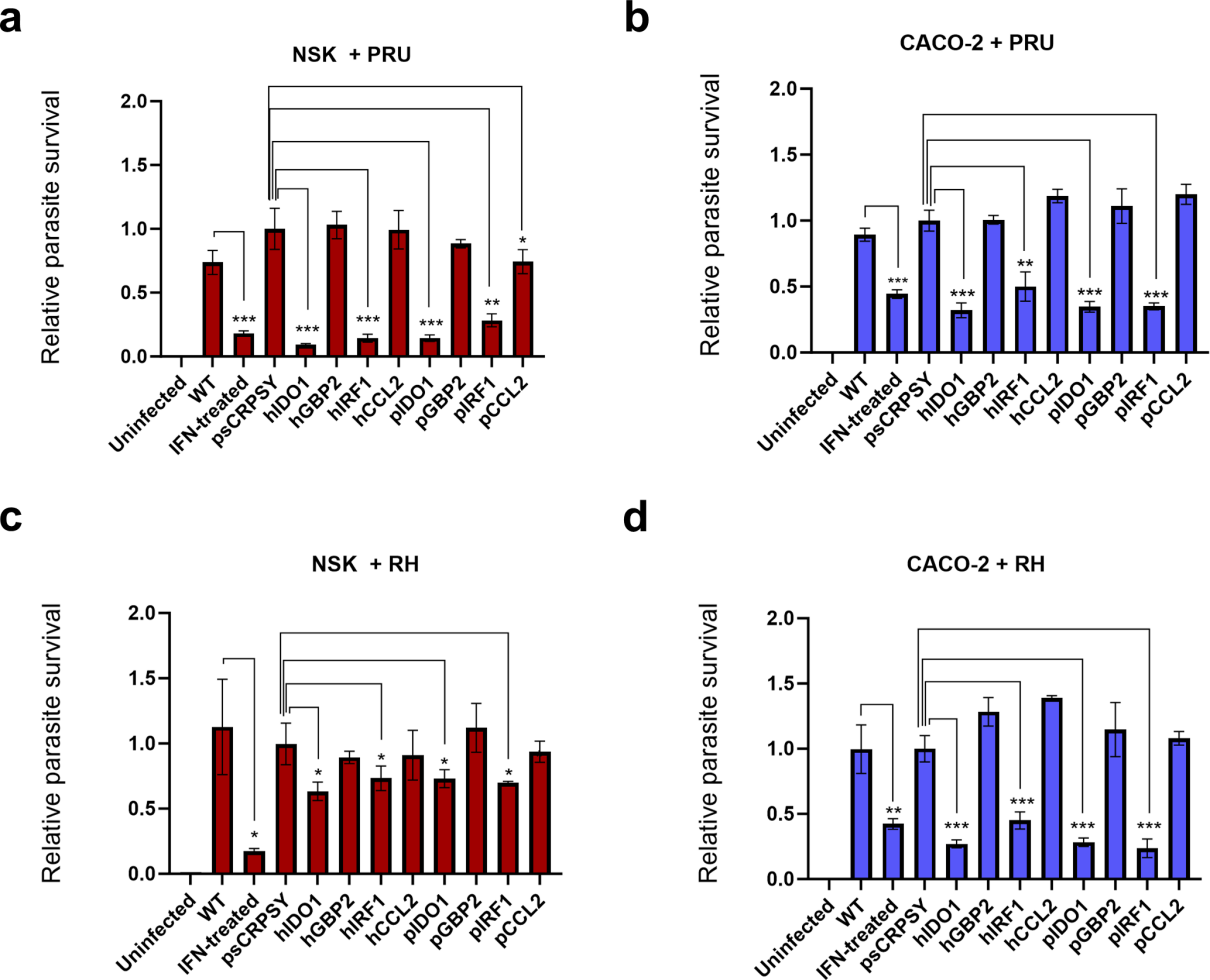
Overexpression of homologous or heterologous IDO1 inhibits *Toxoplasma* growth. **(a–d)** Wild type (WT) or mutant NSK **(a, c)** and CACO-2 **(b, d)** cells expressing individual ISG plasmid or the empty vector were infected with *Toxoplasma* gondii PruA7 **(a, b)** or RH1-1 **(c, d)** at MOI of 0.2 for 72 h or 48 h, respectively. Infected cells were lysed and luciferase activity was measured in each group using Cytation 3 imaging station. Luminescence as parasite burden in each cell line was normalised to cells expressing the empty vector control. n=3, *p<0.05; p<0.005; *p<0.0005; and ns, not significant by unpaired t-test. Data shown from one representative experiment (mean and SEM as error bars) out of 3 biological repeats showing similar trend.

Having established that porcine or human IFNγ cannot inhibit *Toxoplasma* in heterologous human or porcine cells, respectively, we sought to establish if similar *Toxoplasma* pathogenesis in humans and pigs is due to functional conservation of ISGs. Therefore, we performed a mini gain-of-function screen of four human ISGs that are known to control *Toxoplasma* growth, and their porcine orthologues, in porcine NSK and human CACO-2 cells. The ISG expression mini-library comprised of IRF1, which is known to control *Toxoplasma* through transcriptional regulation of other genes (38); a chemokine CCL2 implicated in *Toxoplasma* control (39); IDO1, which degrades L‐tryptophan to deprive the parasite of this essential nutrient (40); and guanylate binding protein 2 (GBP2), that restricts *Toxoplasma* growth (41). To do this, NSK and CACO-2 cells were separately transduced with lentivirus expressing individual ISG plasmid (24) in a one ISG per well format, selected with puromycin and infected with a type II PruA7 *Toxoplasma* strain. Both heterologous and homologous expression of IDO1 and IRF1 significantly inhibited *Toxoplasma* growth in porcine NSK cells (**Figure 2a**). Similarly, the homologous or heterologous expression of IDO1 and IRF1 significantly reduced parasite growth in human CACO2 cells (**Figure 2b**). Similar observations were made with a type I (RH1-1) *Toxoplasma* strain (**Figures 2c, d**). Additionally, we observed a small, but significant reduction in PruA7 growth, but not RH1-1, following homologous expression of porcine CCL2, although human CCL2 did not affect parasite growth in homologous and heterologous cells. However, neither homologous nor heterologous expression of GBP2 affected the growth of PruA7 and RH1–1 in both human and porcine cells. Together, these results suggest a conservation of IDO1 and IRF1 anti-*Toxoplasma* properties between humans and pigs.

### 3.3 Porcine and human IRF1 induce similar sets of ISGs and biological mechanisms in porcine cells

**Figure 3:**
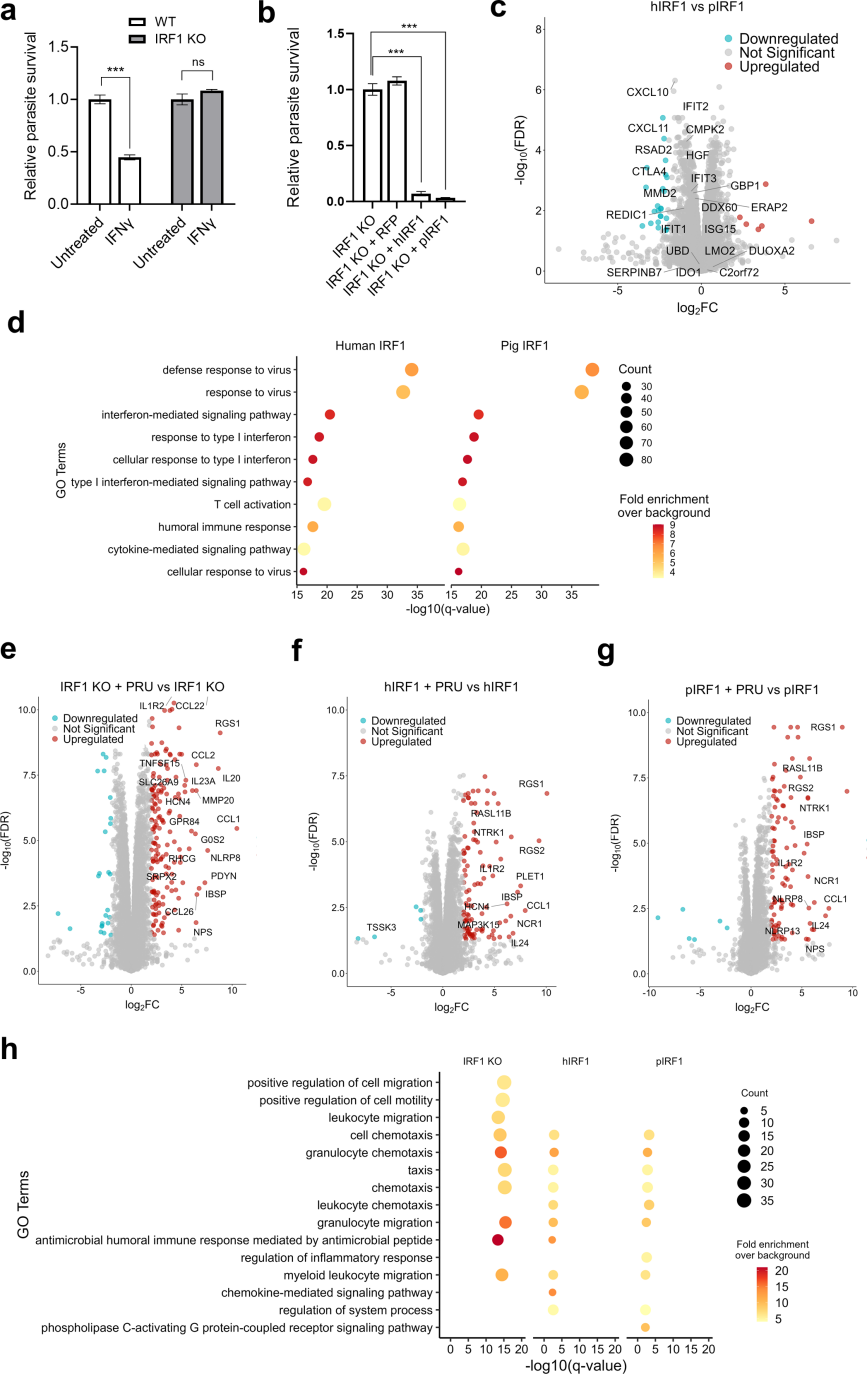
Human IRF1 upregulates anti-*Toxoplasma* genes in porcine cells. **(a)** Wild type (WT) NSK-cas9 and IRF1^-/-^ cells left untreated or stimulated with 50 ng/mL pig IFNγ for 24 h. Cells were then infected with *Toxoplasma* PruA7 at MOI of 0.2 for 48 h. Infected cells were lysed and luciferase activity was measured in each group using Cytation 3 imaging station. **(b)** NSK IRF1^-/-^ cells were left untransduced or transduced with lentivirus expressing empty pSCRPSY, pSCRPSY-hIRF1 or pSCRPSY-pIRF1 plasmid. At 72 h post-transduction, transduced cells were infected at MOI of 0.2 with *Toxoplasma* PruA7 for 48 h. Luciferase activity was measured in each group using Cytation 3 imaging station. Luminescence as parasite burden in each cell line was normalised to untransduced WT control. n=3, *p<0.05; **p<0.005; ***p<0.0005; and ns, not significant by unpaired t-test. Data shown from one representative experiment out of 3 biological repeats showing similar trend. **(c)** Differential gene expression in NSK IRF1^-/-^ expressing hIRF1 relative to pIRF1. NSK IRF1^-/-^ were transduced with lentivirus expressing empty pSCRPSY (control), pSCRPSY -hIRF1 or pSCRPSY-pIRF1 plasmid. Transduced cells were replated at 48 h post-transduction and incubated for 72 h. Cells were harvested, and RNA was isolated from each group, sequenced and analysed. **(d)** Enriched biological processes in IRF1-expressing cells. Gene ontology enrichment analysis was carried out with hIRF1- and pIRF1-upregulated genes (FDR < 0.05 and logFC > 2) using ClusterProfiler. **(e–g)** Differential gene expression in *Toxoplasma*-infected IRF1^-/-^ and IRF1-expressing cells relative to uninfected cells. NSK IRF1^-/-^ were transduced with lentivirus expressing empty pSCRPSY **(e)**, pSCRPSY-hIRF1 **(f)** or pSCRPSY-pIRF1 **(g)** plasmid. Transduced cells were replated at 48 h post-transduction and incubated for further 24 h. Cells were then left uninfected or infected with *Toxoplasma* PruA7 for 48 h. Cells were harvested, and RNA was isolated from each group, sequenced and analysed. Gene expression was compared between infected and uninfected cells in each group. **(h)** Enriched biological processes in *Toxoplasma*-infected IRF1^-/-^ and IRF1-expressing cells relative to uninfected controls. Gene ontology enrichment analysis was carried out with genes upregulated following *Toxoplasma* infection (FDR < 0.05 and logFC > 2) using ClusterProfiler.

The IFN-stimulated transcription factor IRF1, is indispensable for IFN-induced immunity against *Toxoplasma* in mice (42), and human cells (43). Additionally, IRF1 can modulate IFN-independent immunity (20), known to control *Toxoplasma*. In order to determine the impact of IRF1 depletion on *Toxoplasma* control in porcine cells, we generated clonal IRF1^-/-^ NSK cells using CRISPR/cas9 (**Supplementary Figure 2a**). Expectedly, the IFNγ-stimulated IRF1-deficient porcine cells are highly susceptible to *Toxoplasma* (**Figure 3a**), confirming the critical role of IRF1 in porcine IFNγ-mediated immunity to *Toxoplasma*. Interestingly, the ectopic expression of either heterologous human (h) or homologous porcine (p) IRF1 restored *Toxoplasma* inhibition in IRF1-deficient NSK cells (**Figure 3b**). Given that IRF1 controls *Toxoplasma* indirectly by regulating the expression of broad-set of ISGs (38, 44), we determined the transcriptome profile of NSK cells expressing pIRF1 or hIRF1. We observed that complementation of IRF1^-/-^ porcine cells with either heterologous hIRF1 or homologous pIRF1 resulted in the dysregulation of mostly the same set of genes (**Supplementary Figures 2b, c**). A total of 532 genes were upregulated (≥2 Log_2_FC, FDR < 0.05) by human IRF1, compared to 545 genes induced by the porcine IRF1 (**Supplementary Figures 2b–d**; **Supplementary Table 1**) out of which, 458 genes were upregulated by both hIRF1 and pIRF1. We also observed a similar trend when analysing the transcriptome of *Toxoplasma*-infected hIRF1 or pIRF1-complemented cells (**Supplementary Figure 2e**). Notably, the top IRF1-upregulated genes in NSK cells include ISGs with known anti-*Toxoplasma* activity in human cells including IDO1 and GBP1 (**Supplementary Figures 2b, c**; **Figure 3c**). Additionally, gene ontology (GO) enrichment analysis revealed that both hIRF1 and pIRF1 induce the same biological processes (**Figure 3d**). The top IRF1-induced biological processes are related to interferon-mediated signaling pathway and response to type I interferon and humoral immune response that are known to be essential to anti-*Toxoplasma* defense.

We previously reported that *Toxoplasma* infection induces transcriptional responses in infected host cells, and that IFNγ modulates these responses (45). To investigate the impact of IRF1 expression on *Toxoplasma*-induced regulation of porcine cells, we compared the transcriptomes of infected and uninfected porcine IRF1^-/-^ NSK cells expressing empty vector, hIRF1 or pIRF1. Expectedly, *Toxoplasma* infection of NSK IRF1^-/-^ induces transcriptional regulation of immune response genes including *IL20, CCL1* and *CCL2* (**Figure 3e**). Consistent with IFNγ-mediated control of parasite-induced host gene regulation, the complementation of the knockout cells with either hIRF1 or pIRF1 resulted in transcriptional changes in several genes upregulated by *Toxoplasma* in IRF1^-/-^ cells (**Figures 3f, g**), thereby dampening the biological processes induced by the parasite in the absence of IRF1 (**Figure 3h**). Collectively, this shows that hIRF1 can regulate gene expression in porcine cells to control *Toxoplasma* infection, which indicates potential of the pig as an *in vivo* model for studying IRF1-induced human genes that control the parasite.

### 3.4 IDO1-mediated inhibition of *Toxoplasma* growth is conserved between human and porcine cells

**Figure 4:**
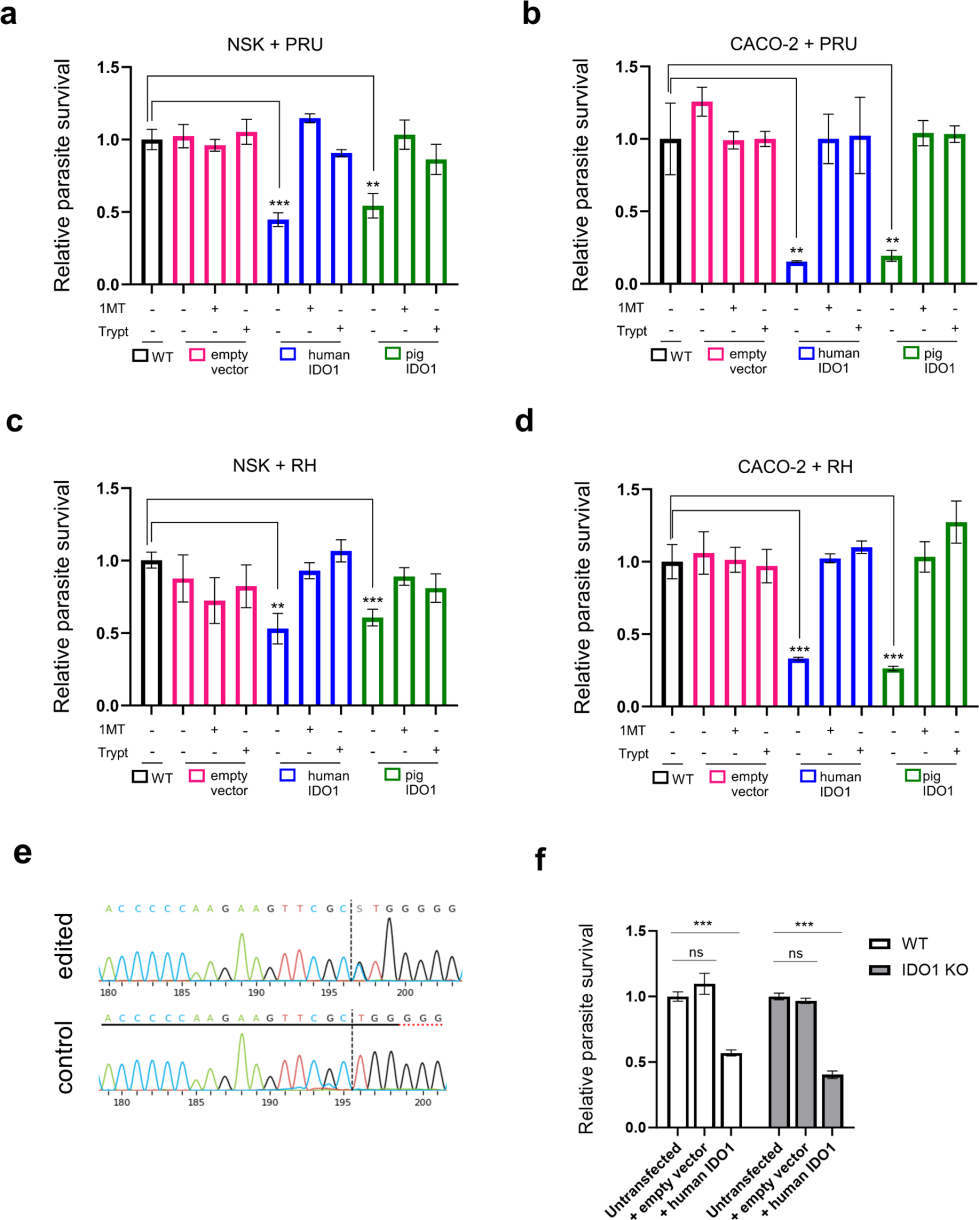
Similar mechanism underlies IDO1 *Toxoplasma* inhibition in human and pig cells. **(A–D)** NSK and CACO-2 cells expressing pSCRPSY, pSCRPSY-hIDO1 or pSCRPSY-pIDO1 were left untreated or treated with 1 mM 1-MT for 24 h. Cells were then were infected at MOI of 0.2 with *Toxoplasma gondii* PruA7 **(a, b)** or RH1-1 **(c, d)** for 1 h. Infected cells were left untreated, treated with 1 mM 1-MT or supplemented with 1 mM 1 tryptophan for 48 h or 72 h for RH1–1 and PruA7 strains respectively. Luciferase activity was measured in each group using Cytation 3 imaging station. Luminescence as parasite burden in each group was normalised to cells expressing the empty vector control. n=3, *p<0.05; **p<0.005; ***p<0.0005; and ns, not significant by unpaired t-test. Data shown from one representative experiment out of 2–3 biological repeats showing similar trend. **(e)** Sequencing confirmed editing of IDO1 in NSK monoclonal population with 98% efficiency. Sequences were analysed using ICE to generate the traces (https://ice.editco.bio/#/). **(f)** NSK IDO^-/-^ cells were left untransduced or transduced with lentivirus expressing pSCRPSY or pSCRPSY-hIDO1. At 72 h post-transduction, transduced cells were infected at MOI of 0.2 with *Toxoplasma* PruA7 for 48 h. Luciferase activity was measured in each group using Cytation 3 imaging station. Luminescence as parasite burden in each cell line was normalised to untransduced WT control. n=3, *p<0.05; ns, not significant by unpaired t-test. Data shown from one representative experiment out of 3 biological repeats showing similar trend.

IDO1 catalyses the rate-limiting step in the degradation of L‐tryptophan along the kynurenine pathway (46), which leads to tryptophan depletion thereby depriving pathogens such as *Toxoplasma* of this essential amino acid. Previous studies have shown that IFNγ-induced inhibition of *Toxoplasma* in human HeLa cells is entirely dependent on IDO1 and can be reversed by tryptophan supplementation or treatment with IDO1 inhibitor, 1-methy tryptophan (1-MT) (47). However, neither tryptophan nor 1-MT reversed IFNγ-induced *Toxoplasma* inhibition in human foreskin fibroblast (HFF) cells, suggesting that dependence on this pathway is cell-type specific in human cells (47). To confirm that the observed inhibition of *Toxoplasma* in human CACO-2 and porcine NSK cells expressing IDO1 was due to IDO-mediated tryptophan deprivation, we pre-treated NSK and CACO-2 overexpressing IDO1 with 1-MT for 24 h before *Toxoplasma* infection. Alternatively, the cells were cultured in media supplemented with tryptophan for 1 h pre-infection. Both 1-MT treatment or tryptophan supplementation reversed the inhibition of *Toxoplasma* PruA7 growth induced by heterologous IDO1 expression in NSK (**Figure 4a**) and CACO-2 (**Figure 4b**) cells. Similar findings were observed against type 1 *Toxoplasma* RH1–1 in CACO-2 (**Figure 4c**) and NSK (**Figure 4d**). To rule out possible induction of endogenous IDO1 following heterologous IDO1 expression, IDO1-knockout NSK cells were generated using CRISPR/cas9 editing (**Figure 4e**) and then complemented with hIDO1. The heterologous expression of hIDO1 resulted in significant reduction in parasite burden in both WT (p= 0.0012) and IDO1^-/-^ (p= 0.0026) (**Figure 4f**). Together these results confirm that heterologous IDO1 expression depletes tryptophan to drive *Toxoplasma* inhibition in human and porcine cells.

The comparable anti-*Toxoplasma* activity between human and porcine IDO1 (**Figure 2**) suggests conservation in IDO1 enzymatic functional domain between the two species. Structurally, human IDO1 consists of 403 amino acids, with a well-conserved fold comprising a large C-terminal domain, which contains nine alpha helices forming the catalytic cleft essential for its enzymatic activity (48). It also has a smaller N-terminal domain, which harbours two functional immunoreceptor tyrosine-based inhibitory motifs (ITIMs), involved in non-catalytic signalling functions (46, 48). A conserved YENM motif bridges these domains (**Supplementary Figure 3**), contributing to post-transcriptional regulation (46, 48). We compared the IDO1 amino acid sequences across species, specifically focusing on man, pig, mouse, rat, cow, and sheep. Notably, pig IDO1 shares approximately 73% sequence similarity with human IDO1, a higher similarity compared to the mouse IDO1, which shows only about 61% similarity (**Supplementary Figure 3**). However, the L-tryptophan binding domain is highly conserved across all the species examined. This includes S167 which receives the indole ring through H-bond, as well as F163 and Y126 which allow subsequent π‐stacking and hydrophobic interactions. We also observed conservation of T379, the binding site of the L‐Tryptophan ammonium group and R231 and T379 which form three hydrogen bonds with the carboxylic moiety (46).

## 4 Discussion

The ability of *Toxoplasma* to infect virtually all vertebrate cells is matched by a somewhat generally conserved requirement for IFNγ-dependent control of the parasite in all vertebrates. IFNγ binding to its receptors activates the Janus-activated kinase (JAK) resulting in the phosphorylation of signal transducer and activator of transcription 1 (STAT1), which homodimerizes and translocates to the nucleus. In the nucleus, STAT1 homodimer binds to gamma-activated sequences (GAS) on the promoter region of genes collectively termed interferon stimulated genes (ISGs) (49). The ISGs inhibit *Toxoplasma* through diverse mechanisms including nutrient deprivation (50, 51), production of reactive oxygen/nitrogen species (52), dissolution of the parasite vacuolar membrane (41, 53), host cell death (47, 54), and further induction of other anti-*Toxoplasma* effector genes (38). Here we show that some of the ISGs mediate conserved anti-*Toxoplasma* mechanisms between human and pigs.

The studies of IFNγ-mediated control of *Toxoplasma* in mice and humans have advanced understanding of innate immunity against the parasite, and in addition, highlighted several differences in *Toxoplasma* restriction mechanisms between humans and mice. For example, a subset of ISGs, immunity-related GTPases (IRGs), are critical to the anti-*Toxoplasma* effect of IFNγ in mice (11), but humans do not express IFN-induced IRGs. It appears that ISGs that inhibit *Toxoplasma* in man are cell-type specific, as seen with IDO1, which is essential to interferon control of the parasite in some cells, but not others (40, 47). Interestingly, the disparate mechanisms of IFN-mediated control of *Toxoplasma* between mouse and human cells have resulted in different parasite counteracting mechanisms. For example, the rhoptry proteins (ROP5 and ROP18), which inhibit IRGs, are essential for *Toxoplasma* virulence strategies in IFN-stimulated murine cells (55), whereas a combination of TgIST and secretory granules GRA16, GRA24, and GRA28 is required for complete inhibition of IFNγ responses in human cells (56). Together, these observations indicate the need for animal models that accurately mimic cell-autonomous response to *Toxoplasma* in humans.

Because *Toxoplasma* pathogenesis in pigs and humans is highly comparable, and IFNγ is required to control *Toxoplasma* infection in both species, we investigated if this comparable pathogenesis is due to conserved IFNγ signalling between the species. We initially stimulated human and porcine cells with heterologous porcine and human IFNγ, respectively, and determined the impact on *Toxoplasma* growth. We observed that neither human nor porcine IFNγ could restrict the parasite in heterologous cells. We then sought to establish if comparable infection outcome is mediated by functionally conserved ISGs. Out of four ISGs tested, the homologous or heterologous expression of IRF1 and IDO1 significantly inhibited *Toxoplasma* growth in both human and porcine cells, suggesting the conservation of anti-*Toxoplasma* properties of these genes between the two species. The human and pig GBP2 did not show anti-*Toxoplasma* activity in both human and pig cells assayed. This is consistent with a previous study by Fisch et al. (41) which showed that depletion of either human GBP1, 2 or 5 in human macrophages abolished IFNγ restriction of *Toxoplasma*, but induction of a single GBP alone failed to restrict parasite growth, suggesting that human GBPs act in concert. A different study showed that GBP1 induction is required to recruit GBP2, 3 and 4 to the surface of *Shigella flexneri*, indicating interdependence among the GBPs (57).

The CCL2 is produced by myeloid cells in response to acute *Toxoplasma* infection, and binds to the chemokine receptor CCR2, thereby recruiting CCR2^+^ monocytes to the site of infection through peripheral circulation (58, 59). Additionally, CCR2^+^ monocytes are recruited to the brain through the blood-brain barrier (BBB) during chronic infection, and are essential to control of cerebral toxoplasmosis (60, 61). The failure of CCL2 to inhibit *Toxoplasma* in porcine NSK and human CACO-2 cells *in vitro* suggests that its role in anti-*Toxoplasma* immunity depends on monocyte recruitment under *in vivo* system. This reiterates previous observations that anti-*Toxoplasma* activity of some human ISGs is exclusive to specific cell types (40, 51, 55, 62), and indicates that similar patterns may underly parasite control in porcine cells.

The essential role of IRF1 in *Toxoplasma* control has been described *in vivo* in mice (42) and in human cells (43). Expectedly, we observed that IRF1-depleted porcine cells failed to control *Toxoplasma*, confirming the essential role of IRF1 in IFN-mediated immunity to *Toxoplasma*. Interestingly, the loss of IFNγ-mediated parasite restriction in IRF1-depleted porcine cells could be reversed by ectopic expression of human IRF1. Consistent with IRF1 transcriptional regulation of other ISGs (44), human and porcine IRF1 induced similar transcriptional profiles in porcine cells, suggesting that porcine models are a suitable surrogate to probe hIRF1-induced transcriptional regulation. A total of 532 genes were upregulated by ≥2-fold in response to hIRF1 expression, compared to 546 genes induced by the pIRF1. Most of the upregulated genes were similar in both groups, and gene-set enrichment analysis revealed that similar processes were enriched by comparable fold at highly similar statistical significance. The induced processes include IFN-mediated signaling pathway, response to type I interferon and defense response to virus, which are largely consistent to previously described IRF1-induced processes in human cells (38). Additionally, the expression of hIRF1 and pIRF1 modulate the *Toxoplasma-*induced transcriptional regulation of porcine cells.

IDO1 inhibits *Toxoplasma* directly through tryptophan degradation in some human cells (40, 51, 55). We found that both homologous and heterologous expression of IDO1 resulted in *Toxoplasma* restriction in pig NSK and human CACO-2 cells, which could be reversed by tryptophan supplementation or pre-treatment with the IDO1 inhibitor, 1-MT, in both cells. This indicates that similar mechanisms through tryptophan depletion underly IDO1 control of *Toxoplasma* in human and porcine cells. To further investigate the role of IDO1 in IFN-control of *Toxoplasma* in pig cells, we depleted IDO1 and observed reversal of IFN restriction of the parasite. Interestingly, the introduction of hIDO1 into the IDO1 deficient pig cells restricts parasite replication, to the same extent as expression in WT, which indicates that hIDO1 can protect pig cells from *Toxoplasma* infection, independent of endogenous pIDO1. This can be explained by the high conservation, especially in the active motifs involved in tryptophan metabolism, between pIDO1 and hIDO1 (46, 48).

This study reports similar IFN-mediated anti-*Toxoplasma* mechanisms through IRF1 and IDO1 in human and pig cells. We found that heterologous human IRF1 expression induced anti-*Toxoplasma* genes and processes in porcine cells, whereas human IDO1 expression could directly inhibit the parasites through tryptophan deprivation in porcine cells. This demonstrates the potential for the use of pig to model *in vivo* studies of human genes that inhibit *Toxoplasma* and other zoonotic pathogens controlled by IFN, which cannot otherwise be carried out in humans. Additionally, it highlights the potential for the development of future ‘one health’ immunotherapies to control zoonotic diseases of man and pig.

Additional studies are needed to determine if IRF1 and IDO1 from other species (e.g., mouse) restrict *Toxoplasma* in homologous and heterologous cells. We will investigate which of the hundreds of orthologous human and porcine IRF1-regulated genes mediate IRF1 control of *Toxoplasma* in future studies.

## Data Availability

The datasets presented in this study are publicly available. This data can be found on NCBI GEO repository with the accession number GSE326196.
