# Supplementary material for: Heterologous expression of interferon-stimulated genes reveals conserved anti-Toxoplasma properties between human and porcine cells
Source: Front Immunol. 2026 Apr 2;17:1790284. doi: 10.3389/fimmu.2026.1790284 (PMC13082979; doi:10.3389/fimmu.2026.1790284)
Supplement: Supplementary file 1 [file DataSheet1.docx]

Supplementary Material

**Supplementary Figures**

**Supplementary Figure 1: Cross-reactivity of porcine and human cells to exogenous IFN**γ.

HFF, HeLa and IPEC-J2 cells were stimulated with indicated concentrations of human (U/mL) or pig IFNγ (ng/mL) for 24 h. Cells were then infected with either *Toxoplasma* *gondii* strain PruA7 or RH1-1 at MOI of 0.2 for 72 h or 48 h, respectively. Infected cells were lysed and luciferase activity was measured in each group using Cytation 3 imaging station. Recorded luminescence reading in each group was normalised to cells expressing the empty vector control as relative parasite survival. Data shows average (mean ± SEM, n=3) of one representative experiment out of 3 biological repeats showing similar trend. *p<0.05; **p<0.005; ***p<0.0005; and ns, not significant by unpaired t-test.

**Supplementary Figure 2**. **Human** **IRF1 upregulates anti-*Toxoplasma* genes in porcine cells.**

A) Sequencing confirmed editing of IRF1 in NSK monoclonal population. Sequences were analysed using ICE to generate the traces (<https://ice.editco.bio/#/>).

B-C) Differential gene expression in NSK IRF1^-/-^ expressing hIRF1 (B) or pIRF1 (C) relative to NSK IRF1^-/-^ control. NSK IRF1^-/-^ were transduced with lentivirus expressing empty pSCRPSY (control), pSCRPSY-hIRF1 or pSCRPSY-pIRF1 plasmid. Transduced cells were replated at 48 h post-transduction and incubated for 72 h. Cells were harvested, and RNA was isolated from each group, sequenced and analysed.

D) Number of genes differentially expressed by hIRF1 and pIRF1

E) Differential gene expression in *Toxoplasma*-infected NSK IRF1^-/-^ expressing hIRF1 relative to pIRF1. NSK IRF1^-/-^ were transduced with lentivirus expressing empty pSCRPSY (control), pSCRPSY-hIRF1 or pSCRPSY-pIRF1 plasmid. Transduced cells were replated at 48 h post-transduction and incubated for 72 h. Cells were harvested, and RNA was isolated from each group, sequenced and analysed.

**Supplementary Figure 3. The amino acid sequence alignment for IDO1 from various species**.

Alpha helices and beta sheets are highlighted in blue and red, respectively, based on the human IDO1 sequence. Purple boxes indicate immunoreceptor tyrosine-based inhibitory motifs, and the green box highlights the YENM motif. The percentage of identity alignment compared to the human sequence is indicated at the end of the sequence.
