# Supplementary figures and images for: Heterologous expression of interferon-stimulated genes reveals conserved anti-Toxoplasma properties between human and porcine cells
Source: Front Immunol. 2026 Apr 2;17:1790284. doi: 10.3389/fimmu.2026.1790284 (PMC13082979; doi:10.3389/fimmu.2026.1790284)

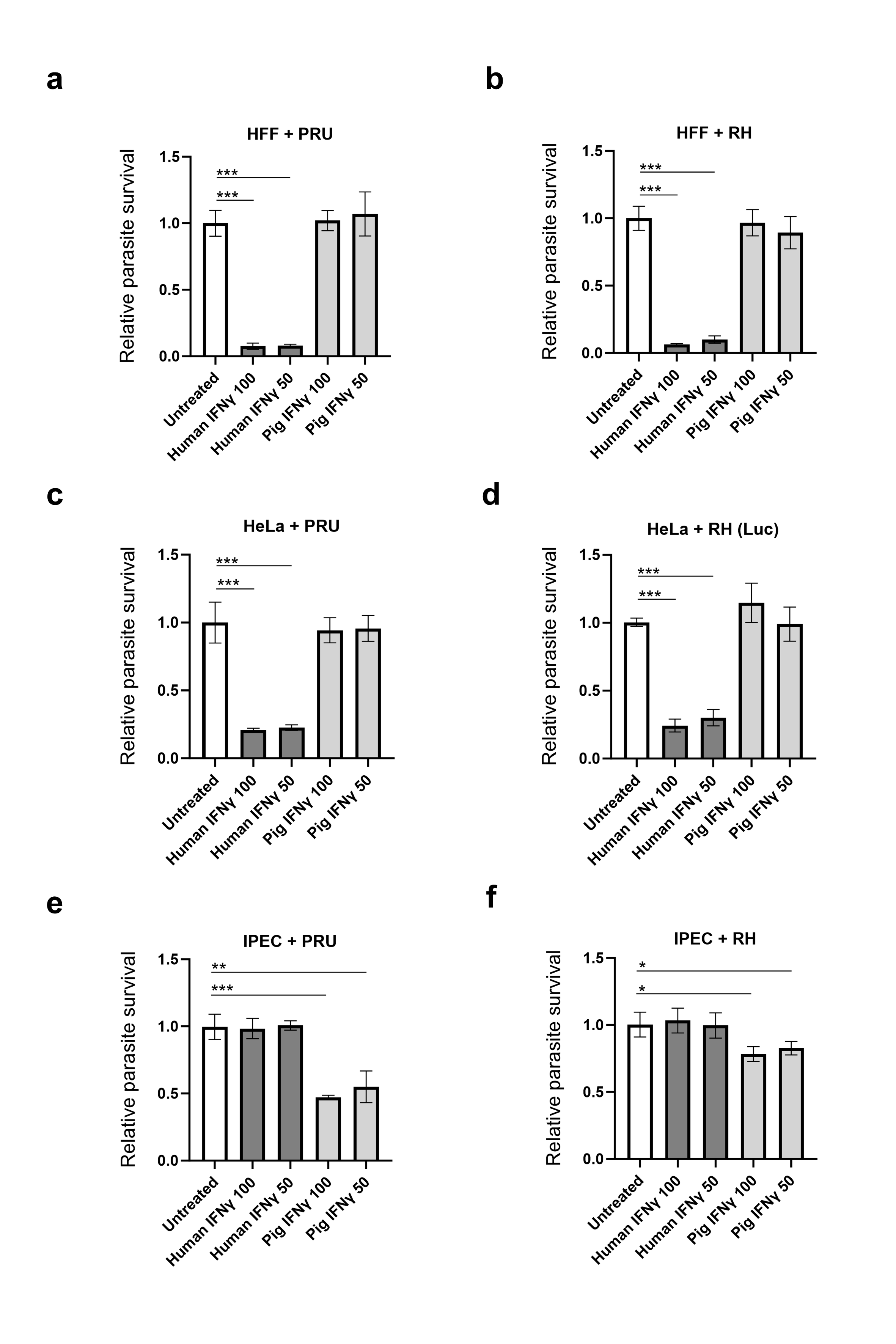

Supplement: Supplementary file 2 [file Image1.tif]

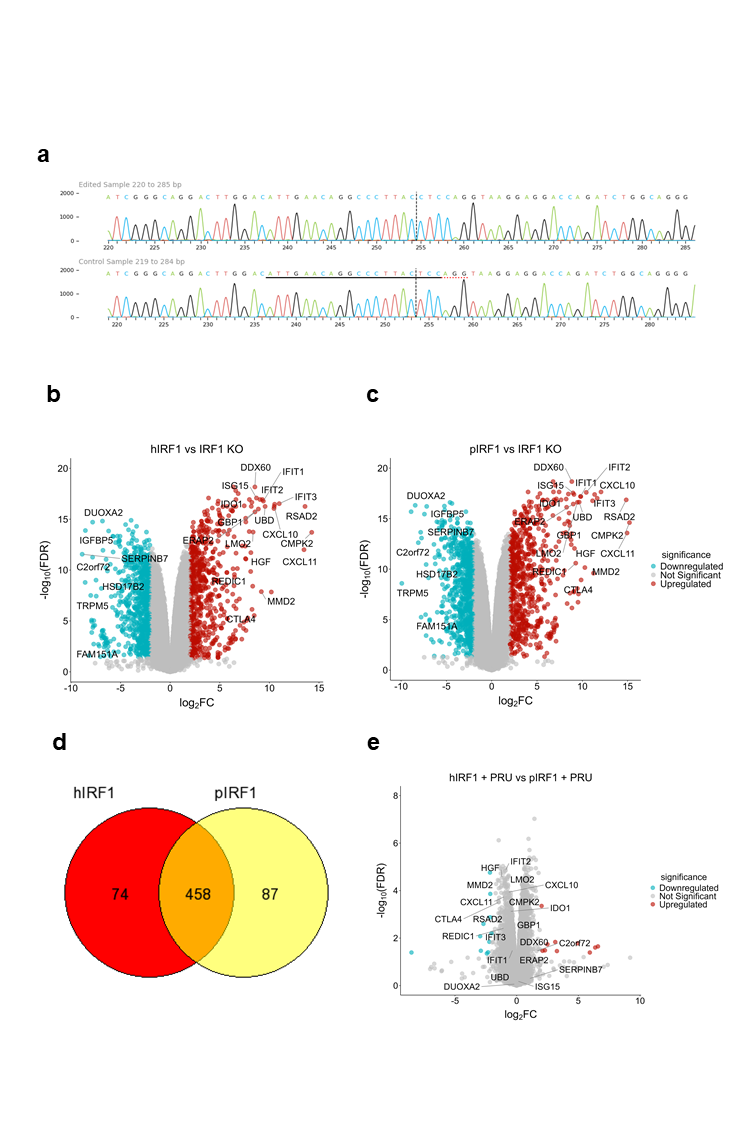

Supplement: Supplementary file 3 [file Image2.tif]

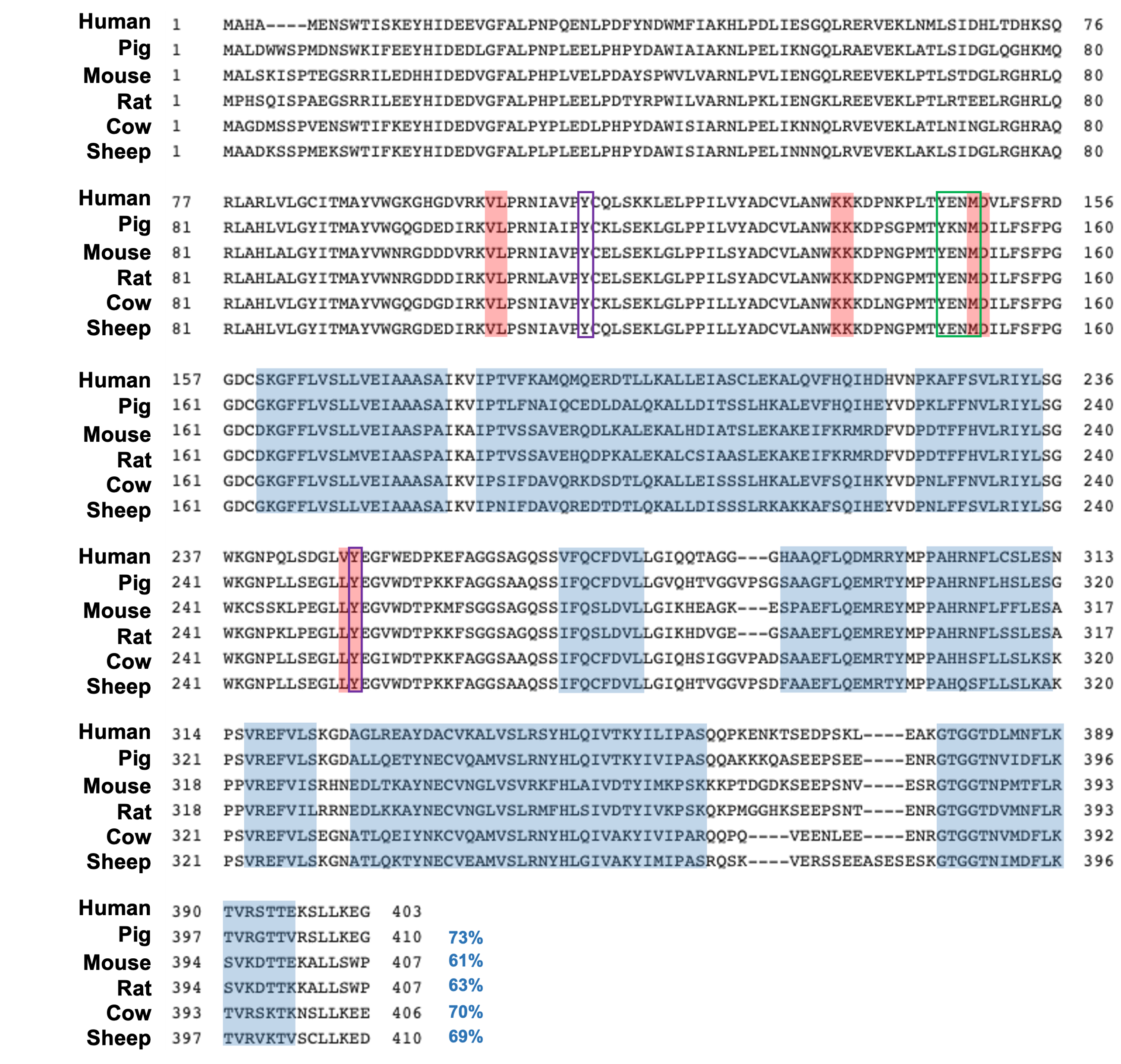

Supplement: Supplementary file 4 [file Image3.tif]
